# Oncogenic role of ERV with therapeutic potential

**DOI:** 10.3389/fimmu.2026.1807944

**Published:** 2026-04-24

**Authors:** Pan-Hui Xia, Wei-Li Zhao, Jie Xiong

**Affiliations:** 1 Shanghai Institute of Hematology, State Key Laboratory of Medical Genomics, National Research Center for Translational Medicine at Shanghai, Ruijin Hospital Affiliated to Shanghai Jiao Tong University School of Medicine, Shanghai, China; 2 Pôle de Recherches Sino-Français en Science du Vivant et Génomique, Laboratory of Molecular Pathology, Shanghai, China

**Keywords:** cancers, endogenous retroviruses, epigenetic modification, oncogenesis, therapeutic targets

## Abstract

Endogenous Retroviruses (ERVs) are originated from ancient exogenous viruses, which integrate into the host genome after infection and persist across all vertebrate lineages. Complete ERV consists of viral genes (*gag*, *pro*, *pol*, and *env*) in the center and two Long Terminal Repeats (LTRs) at both ends, which encode viral structural proteins, critical enzyme, and glycoprotein. ERVs constitute approximately 8% of human genome and function in a wide range of physiological and pathological processes, including embryonic development, inflammation and infection, neurodegenerative diseases, cancers, etc. The expression of ERVs is controlled mainly by epigenetic modification, transcriptional regulation and post-transcriptional modulation, which offered important therapeutic targets. In this review, we reviewed the structure and function of ERVs, summarized recent research advances on how ERVs contribute to cancer initiation and progression, and introduced some therapies targeting ERVs.

## 1 Introduction

Transposable elements are mobile DNA elements that are universally interspersed in eukaryotic genomes, which can be divided into DNA transposons and retrotransposons according to the mechanism of movement (1). DNA transposons use “cut-and-paste” mechanism to move themselves to exert a regulatory activating role. Retrotransposons, which are classified into LTR and non-LTR retrotransposons based on the presence or absence of LTRs at their termini, use RNA as transposition intermediate to copy and integrate the chromosome into the genome (2, 3). The overwhelming majority of LTR retrotransposons are ERVs, also known as endogenous retroelements, originating from ancient exogenous viruses and integrating into the host genome after infection (4–6). ERVs persist across all vertebrate lineages, though their genomic abundance exhibits significant interspecies variation. For example, in the human genome, ERVs constitute approximately 8%, while in mice this proportion rises to around 10%, and in dogs and pigs it decreases to about 4% (7).

Driven by concerns for human health and the human genome, extensive research has been conducted in Human Endogenous Retroviruses (HERVs). In recent years, growing evidence has revealed that HERVs are closely linked to placental development, immune response, cancer development, and other biological processes. Moreover, HERVs also play significant roles in transcriptional regulation and evolutionary change (8). This review dedicates special attention to biological function and pathological mechanism of HERVs in the context of multiple cancers (**Table 1**), paving novel therapeutic avenues for anti-tumor therapies.

**Table 1:** Pathogenic role of ERV in cancers.

List of ERVs	Pathogenic role	Molecular mechanism	Cancer type	Reference
HERV-K env	Oncogenic factor	activate ERK1/2 signaling, induce expression of ETV4, ETV5, and EGR1 knockdown of HERV-K env upregulate p53, downregulate EGFR, NF-kB, Ras, p-RSK, and blockade Ras/Raf/MEK/ERK signaling pathway	Breast cancer	Lemaître et al., 2017 (32); Zhou et al., 2016 (33)
HERV-K transcript	Oncogenic factor	TROJAN ubiquitinate ZMYND8 to release metastasis suppression	Breast cancer	Jin et al., 2019 (81)
HERV-W env	Oncogenic factor	combination with syncytin-1 and ASCT-2 to promote the fusion of breast cancer cells with endothelial cells	Breast cancer	Gao et al., 2021 (82)
HERV-K Rec	Oncogenic factor	bind with TZFP and androgen receptor to activate c-myc	Testicular cancer	Kaufmann et al., 2010 (73)
ERV9 LTR	Tumor suppressor	induce the expression of GTAp63	Testicular cancer	Beyer et al., 2011 (39)
HERV-W env	Oncogenic factor	Knockdown of HERV-K env affects the expression of RB and CyclinB1	Ovarian cancer	Ko et al., 2022 (83)
HERV-K Rec	Oncogenic factor	binds to the human small glutamine-rich tetratricopeptide repeat protein to activate androgen receptor	Prostate cancer	Hanke et al., 2013 (74)
HERV-K transcript	Oncogenic factor	form new gene fusions with transcription factor ETS-related genes (e.g., ETV1) and encode fusion proteins	Prostate cancer	Hermans et al., 2008 (34)
HERV-K env	Oncogenic factor	mediate intercellular fusion and promote nuclear atypia	Melanoma	Huang et al., 2013 (75)
HERV-L transcript	Oncogenic factor	activate PI3K-AKT, MAPK, TNF and NOTCH signaling pathways and the expression of BTK	Leukemia	Ferlita et al., 2023b (77)
HERV-K Np9	Oncogenic factor	activate such as β-catenin, ERK, c-myc, AKT, and Notch1 signaling pathways	Leukemia	Chen et al., 2013 (35)
HERV-K Np9 and Rec	Oncogenic factor	interact with tumor suppressor promyelocytic leukemia zinc finger protein to activate c-myc	Leukemia	Denne et al., 2007 (78)
LTR2	Oncogenic factor	form the APOC1-LTR2 element and functions as enhancer of the APOC1	Leukemia	Deniz et al., 2020b (37)
LTR	Oncogenic factor	Activate the colony-stimulating factor 1 receptor gene induce the overexpression of the pro-inflammatory transcription factor interferon regulatory factor5	Lymphoma	Lamprecht et al., 2010 (61), Babaian et al., 2016 (36)
HERV-W	Oncogenic factor	Syncytin-1 mediate membrane fusion and transfer tumor cell signals	Lymphoma	Laukkanen et al., 2020 (80)
HERV-K	Oncogenic factor	activate transcription factors (e.g., RUNX3, EBF1, and EBNA2) and enhance their targeted genes expression (e.g., CSF1R, IRF5)	Lymphoma	Leung et al., 2018 (63)
LTR2	Oncogenic factor	transcribe FABP7 gene chimeric transcripts (LTR2-FABP7) and encode the FABP7 protein heterodimer	Lymphoma	Lock et al., 2014 (64)

## 2 HERV structure

Complete HERV consists of viral genes (*gag, pro, pol*, and *env*) in the center and two LTRs at both ends, which is similar as the integrated proviral structure of exogenous retroviruses (7). These canonical viral genes each performs its designated role, for example, *gag* encodes viral structural proteins, *pro* and *pol* encode critical enzyme, and *env* encodes the glycoprotein (9, 10). LTRs contain not only promoter and enhancer elements but also many gene loci available for transcription factor binding (11). Due to recombination of the LTRs of complete elements, over 80% of the HERVs are composed of solo LTR (12). As an important site for epigenetic modifications, LTR play an important role in controlling the expression of HERVs (13).

ERVs are classified into three classes according to the sequence similarities of *pol* gene with retroviruses (7). For HERVs, the sequences of class I HERVs are closely related to the γ-retroviruses, class II HERVs to β-retroviruses, and the class III HERVs to the spumaviruses (14). Moreover, HERVs can be further classified and named by tRNA which binds primer binding site to initiate reverse transcription (e.g., HERV-K, HERV-W, HERV-H) (15). For example, HERV-K means using lysine tRNA to prime reverse transcription, which is the most recently integrated and active group of human proviruses (16). HERV-K (HML-2) proviruses are classified into two types, type I and type II, according to the presence or absence of a 292-bp deletion. The type I expresses the Np9 protein, while type II expresses the Rec protein (17). Currently, there is extensive research on HERV-K (HML-2), and its role in cancer will be emphatically discussed in the subsequent section on oncology.

## 3 HERV expression

Most HERVs lost transcriptional ability under physiological conditions, largely due to host epigenetic repression and mutations acquired throughout evolution (18). However, accumulating evidence suggests that these regulatory mechanisms can be disrupted in various diseases, resulting in aberrant expression of HERVs. For example, HERV-K proteins have been detected in testicular germ cell tumors for many years. Particularly, gene expression of HERV-K is detected in carcinoma *in situ*, comparing with cells of the testis (19). Besides, six HERV-W loci were also overexpressed in testicular cancer (20). Moreover, HERV-K *env* mRNA was significantly elevated in ovarian epithelial tumors, as compared to normal ovarian tissues. In patients with primary ovarian cancer, HERV-K transcripts and env protein were overexpressed in cancer cells and ascites. HERV-K antibodies were detected in patient sera (21). Overexpression of HERV-K (HML-2) mRNA was detected in prostate cancer and in the blood of chronic myeloid leukemia and acute myeloid leukemia (AML) patients (22). Particularly, the expression of *gag* mRNA is higher in leukemia patients’ PBMCs than normal persons (22). In Hodgkin's lymphoma (HL), HERV-K viral particles are detected in the blood of patients compared to healthy individuals. Meanwhile, HERV-K and HERV-H transcripts can be detected in Hodgkin’s lymphoma cell lines (23, 24).

However, a substantial number of HERVs are expressed in normal tissues (especially in the testis and brain), and the expression is closely linked to histone modifications (e.g., H3K27ac, H3K36me3) (18). Moreover, their expression is associated with biological factors including sex, ethnicity, and age (18). Particularly, the expression of HERVs is essential in biological process of early embryonic development (13). Therefore, HERV expression may represent a double-edged sword, potentially assuming complex dual roles in physiological and pathological contexts. Under pathological conditions, the aforementioned regulatory system is destructed, leading to the reactivation of HERVs, which is associated with the pathogenesis of multiple sclerosis (MS), amyotrophic lateral sclerosis (ALS) and cancers (6).

## 4 Biological and pathological function of HERV

HERVs and their gene products play an indispensable role in the development and function of host cells after years of domestication, which refers to the process of HERVs invading the human genome and transforming into genetic elements beneficial or essential to the host over a prolonged evolutionary process. Syncytin-1 and Syncytin-2, derived from HERV-W *env* and HERV-FRD *env* respectively, are key fusion proteins in placental development, playing a central role in the formation and maintenance of the syncytiotrophoblast, as well as immune regulation and substance exchange between the mother and fetus (12, 25, 26). Besides, the transcriptional repression of HERV-K (LTR5Hs) and HERV-H is recused during the genome-wide demethylation window of early embryonic development (13). Subsequently, the expression of HERV-K-encoded accessory protein Rec and HERV-H-derived long non-coding RNA are induced by recruitment of transcription factors OCT4 and NANOG through their LTR regions, which regulate host gene expression and stabilize the pluripotency network (13). Besides, demethylation of HERVs generates double-stranded RNAs (dsRNAs), which stimulate the production of interferon and innate immune responses (27).

Aberrant expression of HERVs also critically involves in neurodegenerative diseases, such as MS and ALS. MS is a demyelinating disease of the central nervous system. HERV-W envelope is associated with the progression and severity of MS, which enhances the production of inflammatory cytokines and contributes to demyelination and neurodegeneration (28). ALS is characterized by progressive loss of motor neuron function and respiratory muscle dysfunction with extremely low five-year survival (29). HERV-K *env* is overexpressed in the serum and cerebrospinal fluid of ALS patients, interacting with motor neuron degeneration marker TAR DNA-binding protein 43, suggesting a potential role in the pathogenesis and development of ALS (30).

**Figure 1:**
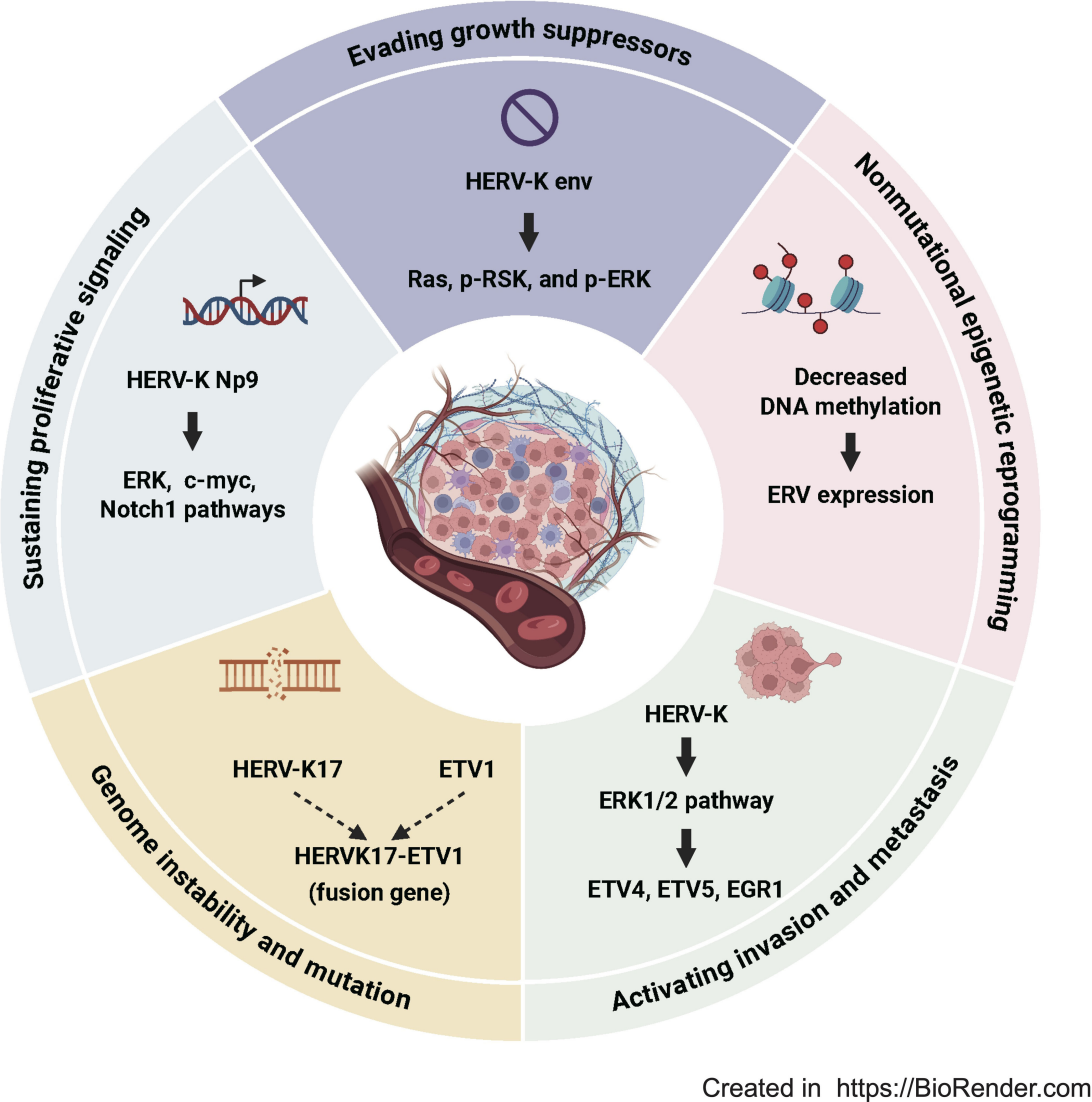
HERVs regulate the hallmarks of cancer. According to the conceptualization of the hallmarks of cancer, HERVs are involved in five key mechanisms: evading growth suppressors, nonmutational epigenetic reprogramming, activating invasion and metastasis, sustaining proliferative signaling and genome instability and mutation.

The conceptualization of the hallmarks of cancer showed that the transformation of human cells from a normal growth state to malignant tumors is facilitated by 14 core capabilities, namely evading growth suppressors, nonmutational epigenetic reprogramming, avoiding immune destruction, enabling replicative immortality, tumor-promoting inflammation, polymorphic microbiomes, activating invasion and metastasis, inducing or accessing vasculature, senescent cells, genome instability and mutation, resisting cell death, deregulating cellular metabolism, unlocking phenotypic plasticity, sustaining proliferative signaling (31). HERVs are primarily involved in several key mechanisms: evading growth suppressors, nonmutational epigenetic reprogramming, activating invasion and metastasis, sustaining proliferative signaling and genome instability and mutation (**Figure 1**) (32–35). Activated HERVs are involved in regulating the expression of pro-oncogenic genes and related pathways, ultimately promote tumor progression and metastasis (36, 37). For example, overexpression of HERV-K (HML-2) has been detected in many cancers, including breast cancer, ovarian cancer, colon cancer, pancreatic cancer, and lymphoma (38), promoting tumor cell proliferation and invasion. Interestingly, suppression of ERV9 LTR interfere genes critical for maintaining cellular homeostasis (e.g., GTAp63), thereby facilitating carcinogenesis (39, 40). These paradoxical findings suggest a complex role of HERVs in cancer, which might be shaped by multiple factors, including the specific HERV locus, the nature of the transcript or protein product, the context of malignant cells and the tumor microenvironment. In breast cancer, HERV-K env activates ERK1/2 signaling and its downstream transcription factors ETV4, ETV5, and EGR1, thereby promoting tumor invasion (32). In testicular cancer, ERV9 LTRs inserted upstream of p63 drive the expression of GTAp63 and thereby contribute to its tumor-suppressive function (40).

Whether the abnormal expression of HERVs is a parallel feature of epigenetic alteration or a key driver of oncogenes remains controversial. Activation of HERV-K induces senescence-like features in human mesenchymal progenitor cells (41), proposing an oncogenic driver. HERV-K (HML-2) *env* triggers epithelial to mesenchymal transition (EMT) of breast epithelial cell line and enhances tumor aggressiveness and metastasis (32). Knockdown of HERV-K suppresses tumor cell proliferation, invasion, and other malignant behaviors in breast cancer cells (33). Besides, overexpression of HERV-K *env* in melanoma cells promotes tumor progression (42). However, HERVs could serve as passenger events secondary to cancer-associated epigenetic dysregulation. For example, in head and neck squamous carcinoma tissues, HERVH and HERV17 families exhibit significant methylation loss compared to non-neoplastic tissues (43). In ovarian cancers, malignant tissues exhibited reduced methylation levels of CpG dinucleotides in both L1 and HERV-W elements, contributing to elevated expression levels (44). Increased promoter activity and reduced methylation levels in the 5’LTR region may be the reasons for elevated HERV-K expression in melanoma (45). Despite the critical association between HERVs and cancer, the expression of HERVs is critically regulated by epigenetic modification, which is a hallmark of cancer (46).

In addition to ERVs, LTR retrotransposons are also involved in multiple pathobiological processes. For example, the Sushi-ichi retrotransposon-derived PEG10 gene is essential for embryonic development, whose aberrant expression is also linked to the proliferation and metastasis of breast cancer, melanoma (47). As a mechanism of action, PEG10 Gag protein can form virus-like particles and bind to cellular RNA Hbegf (Heparin-binding EGF-like growth factor), regulating capillary network and placental development (48, 49). PEG10 enhances the progression of hepatocellular carcinoma by upregulate the anti-apoptotic protein Bcl-2 and downregulating the pro-apoptotic protein Bax (50, 51). PEG10 promotes migration and invasiveness of pancreatic cancer by activating the ERK/MMP7 signaling pathway (52).

According to the International Agency for Research on Cancer, cancer-related deaths account for nearly one-sixth of all global deaths (16.8%) (53). Approximately 20 million new cancer cases have been diagnosed worldwide in 2022, positioning a significant threat to global public health systems and socioeconomic stability (53). Despite the comprehensive investigation on cancer biology, the oncogenic role of HERVs as well as their therapeutic potential are of great interest.

## 5 Mechanisms of HERV in cancer

### 5.1 Epigenetic activation

**Figure 2:**
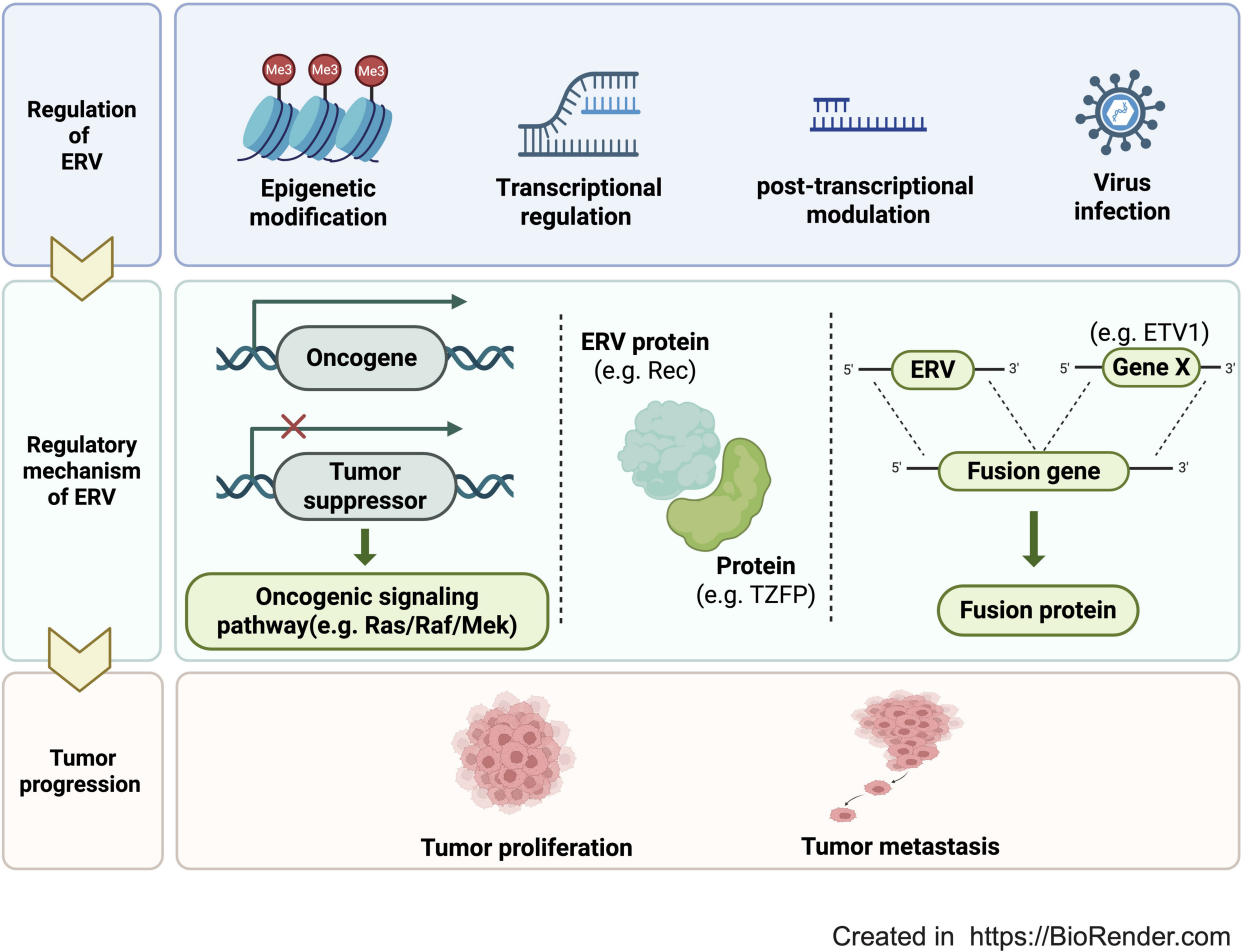
How ERVs contribute to cancer initiation and progression. The expression of HERVs is predominantly regulated by virus infection, transcriptional factors, post-transcriptional modulation, and epigenetic modification. HERVs promote tumor proliferation and metastasis by directly regulating oncogenes, tumor suppressors, and oncogenic signaling pathways, encoding proteins to interact with other functional proteins, as well as fusing with host genes to form fusion proteins.

HERVs are systematically regulated through a complex and sophisticated multi-layered regulatory system (**Figure 2**), primarily involving epigenetic modification, transcriptional regulation and post-transcriptional modulation (25). In normal tissues, epigenetic regulation suppress HERV expression to maintain genome stability and integrity (13). During oncogenesis, global or local hypomethylation of DNA contributes to the upregulation of some HERVs (13). Krüppel associated box zinc finger proteins (KRAB-ZFPs) represent a class of transcription factors, which restrain HERVs expression through forming co-repressor complex with KRAB-associated protein 1(KAP1) and subsequent recruiting histone methyltransferase SETDB1 (15). METTL3 and METTL14-mediated m6A methylation of RNA provides a protective effect in restricting ERV expression and maintaining cellular integrity (54). As small silencing RNAs, Piwi-interacting RNAs (piRNAs) recognize the target ERV transcripts through base-pair complementarity, leading to their cleavage and degradation (55). CpG dinucleotides methylation of retrotransposons, such as HERV-W, controls their expression levels, which were increased in malignant ovarian tissues, compared to non-malignant ovarian tissues (44). HERV-K_22q11.23 provirus expression is significantly increased in androgen-responsive prostate cancer cells due to decreased levels of DNA methylation in the LTRs (56). HERV-K_22q11.23 gag expression is induced by promoter region demethylation and androgen stimulation (57).

Transcription factors also have a role in the activation of HERVs. For example, the combined expression of transcriptional factors Oct4, Nanog, and Sox2 markedly enhances their transactivation capability to upregulate HERV-K LTR5Hs (58). Moreover, the importance of estrogen and progesterone in the development of breast cancer is well established, with estradiol acting by binding to progesterone-response element and stimulating progesterone receptor (PR) expression. PR functions synergistically with octamer-binding transcription factor4 to bind with LTR of HERV-K and activate its expression (59). As a mechanism of action, Melanocyte inducing transcription factor (MITF)-binding motifs (MITF-1, -2, and -3), TATA box (793TATA), and the Inr site (Inr826) function as promoter or enhancer to activate the transcription of HERV-K LTR in melanoma (60). Activation of the colony-stimulating factor 1 receptor gene is prevalent in the Hodgkin lymphoma, the transcriptional promoter of which begins at the LTR element of the MaLR family. Deletion of the corepressor CBFA2T3 and activation of NF-κB lead to demethylation of this LTR, which in turn promotes lymphoma progression (61).

Besides, HERVs also function as transcriptional regulators in multiple cancers. In testicular cancer, the human male germ cell-encoded TAp63 protein (also known as GTAp63) is exclusively expressed in the human testis and functions as a tumor suppressor via protecting the genomic integrity of the male germ line. The human ERV9 LTRs frequently insert into the upstream of p63 and act as a promoter to induce the expression of GTAp63 and maintain the testicular homeostasis. In patients with testicular cancer, silence of ERV LTRs suppresses the expression of GTAp63, which could therapeutically be targeted by histone deacetylases inhibitors to restore the expression of GTAp63, then upregulate TNFRSF10B expression, induce tumor cell apoptosis and inhibit tumor proliferation (39, 40). What’s more, in seminomas, the DNA demethylation of the ERVWE1 promoter induces the derepression of syncytin-1, thus exerting potential pathogenic effects (62). In Leukemia, several families of ERVs, including LTR2B, LTR2C, LTR5B, LTR5_Hs, LTR12C, and LTR13A, bind with key transcription factors related to AML to exert regulatory activity. LTR2 locates upstream of Apolipoprotein C1 (APOC1) forming the APOC1-LTR2 element, which functions as enhancer of the APOC1 and promotes cell proliferation (37). In lymphoma, transcriptional activation of the endogenous retrovirus LOR1a LTR induces the overexpression of the pro-inflammatory transcription factor interferon regulatory factor5, which functions as an oncogene in the development of HL (36). EBV-induce DNA hypomethylation activates the expression of LTRs (e.g., HERVK-18) (63), which act as enhancers and promoters to activate transcription factors (e.g., RUNX3, EBF1, and EBNA2), enhance their targeted genes expression (e.g., *CSF1R*, *IRF5*, *HUWE1*/*HECTH9*), and drive the malignant transformation of EBV-associated B-cell lymphoma (63). In diffuse large B-cell lymphoma (DLBCL), demethylation of LTR2 promoter region leads to the transcription of FABP7 gene chimeric transcripts (LTR2-FABP7) and encode the FABP7 protein heterodimer, which promotes the proliferation of DLBCL (64).

### 5.2 Immune modulation

As an alternative mechanism of action, stress signal such as virus infection also play a critical role (**Figure 2**). For example, exogenous virus infection, such as human immunodeficiency virus 1 and hepatitis B virus activates expression of HERVs and subsequently induces antiviral responses (65, 66). For example, elevated expression of HERV-K Np9 and HERV-R *env* mRNA is detected in the peripheral blood of pediatric acute lymphoblastic leukemia patients, compared with the healthy group, potentially due to viral infections (e.g., herpes simplex virus) -induced HERV trans-activation (67).

The expression of HERVs is precisely modulated by regulatory feedback loop involving innate immunity factors. HERV-derived single-stranded RNA and dsRNAs are recognized by pattern recognition receptors (PRRs) including endosomal PRRs and cytosolic PRRs, to activate innate immune responses, as well as induce the production of type I interferon and inhibit HERV expression (68). In correspondence to its important physiological functions, aberrant expression of HERVs is involved in the pathogenesis of autoimmune diseases and neurodegenerative diseases. Uncontrolled expression of HERVs may lead to continuous production of inflammatory cytokines, contributing to chronic inflammation, tissue damage and autoimmune diseases (17). For example, the expression of HERV-K (HML-2) could enhance inflammatory factors and activate immune response, contributing to immune diseases and inflammatory diseases such as systemic lupus erythematosus and rheumatoid arthritis (17).

In addition, the presence of elevated HERVs-related antibodies and transcripts in patients may indicate that HERV activation is associated with immune recognition. For example, in the sera of patients with early-stage breast cancer, high expression level of HML-2 mRNA, as well as high titers of antibodies against Np9 and Rec proteins (encoded by HERV-K) were associated with increased risk of cancer metastasis (69). ERV3 and HERV-E, as well as the corresponding antibody and env protein are highly expressed in ovarian cancers (70). Patients with ovarian clear cell carcinoma presented with increased expression of HERV-K and HERV-E, which are correlated with inferior prognosis and short survival time (71). Moreover, HERV-K *gag* antibodies are increased in prostate cancer patients with advanced stages and predict poor prognosis (57).

### 5.3 Signaling pathways

HERVs contribute to tumor progression through modulating key signaling pathways that regulate cell survival, proliferation, migration, and invasion. Most signaling pathways are shared by different cancers with varied molecular mechanisms.

HERVs can directly modulate oncogenic signaling pathways. In breast cancer, HERV-K *env* is highly expressed in breast cancer tissues, comparing with normal breast tissues, and participates in signaling pathways of cell survival, proliferation and migration (38, 72). Mechanically, HERV-K *env* activates ERK1/2 signaling and induces the expression of downstream transcription factors ETV4, ETV5, and EGR1, contributing to EMT, tumor invasion and metastasis (32). Knockdown of HERV-K *env* resulted in up-regulation of *p53*, down-regulation of EGFR and NF-kB, and Ras, p-RSK, and p-ERK, as well as blockade of Ras/Raf/MEK/ERK signaling pathway, which inhibit tumor formation, proliferation, transformation, invasion and migration (33) (**Figure 2**). Moreover, HERV-K Np9 protein is also highly expressed in leukemia, which activates multiple leukemic stem/progenitor signaling pathways (such as β-catenin, ERK, c-myc, AKT, and Notch1) to promote the growth and proliferation of leukemic cells (35). In Leukemia, according to RNA sequencing data of B cells in patients with chronic lymphocytic leukemia (CLL), HERV-K, ERVL-E, HERV-H and HERV-L are also highly expressed (77). HERV-L_17p11.2b, which is significantly up-regulated in CLL, is associated with the activation of PI3K-AKT, MAPK, TNF and NOTCH signaling pathways and the expression of BTK (Bruton’s TyrosineKinase), which is a key kinase in the B-cell receptor signaling pathway (77).

HERVs can interact with transcriptional regulators or receptors to regulate signaling pathways. In prostate cancer, Rec, encoded by HERV-K, binds to the human small glutamine-rich tetratricopeptide repeat protein and activates androgen receptor, which promotes cancer progression (74). In testicular cancer, mice models trans genetically expressing HERV-K Rec present with increased chance of developing testicular carcinoma *in situ* (73). HERV-K Rec forms a complex with testicular zinc-finger protein (TZFP), a protein expressed in the testis to repress transcription, and androgen receptor to activate c-myc and promote carcinogenesis (73) (**Figure 2**). HERV-K Np9 and Rec interact with tumor suppressor promyelocytic leukemia zinc finger protein to transcriptionally activate c-myc proto-oncogene and promote carcinogenesis (78).

Besides, HERV-K and HERV-W are highly expressed in blood of cutaneous T-cell lymphomas (CTCL) patients of mycosis fungoides and Sézary syndrome subtype, as compared with healthy persons (79). Syncytin-1 encoded by HERV-W can be detected in CTCL-derived extracellular vesicles, which may mediate membrane fusion and transfer tumor cell signals (80).

### 5.4 Others

In breast cancer, it was also found that multiple HERV-K transcripts were overexpressed, such as HERV-K_17p13.1 in ER+, HER2+, and triple-negative breast cancer (TNBC). Besides, the dominant transcript of LTR70, namely TROJAN, is highly expressed in TNBC, compared with normal breast tissues (81). TROJAN promotes the proliferation and invasion of TNBC by ubiquitinating ZMYND8, a repressor of genes associated with cancer metastasis (81). Additionally, syncytin-1 produced by HERV-W *env* binds with ASCT-2 and promote the fusion of breast cancer cells with endothelial cells, which may contribute to cancer invasion and metastasis (82).

In ovarian cancer, knockdown of HERV-K *env* in ovarian cancer cell lines using CRISPR-Cas9 technology affects the expression of RB and CyclinB1, as well as inhibits the proliferation, migration and invasion of cancer cells (83).

In prostate cancer, the expression of HERV-K_22q11.23 and HERV-K17 are regulated by androgens, and function through fusing with transcription factor ETS-related genes (e.g., *ETV1*) to form new gene fusions (e.g., *HERVK17-ETV1*), encoding novel ETV1 proteins (e.g., dETV1), which promote tumor invasion and migration (34) (**Figure 2**).

In melanoma, there has been abundant evidence revealing HERV-K *pol, gag* and *env* mRNA, as well as gag, env, Rec and Np9 proteins (22), contributing to its malignant transformation. The overexpression of HERV-K *env* mRNA and protein will promote melanoma progression, while HERV-K Rec will maintain the proliferative state of melanoma and prevent melanoma developing to invasive stages (42). HERV-K env is involved in mediating intercellular fusion of melanoma cells and promotes the development of Nuclear atypia, which in turn enhances melanoma cell survival and promotes worsening tumor progression (75). HERV-K env protein is homologous to human oxygen responsive element binding protein, and it may inhibit the formation of redox-enzymes and increase toxic free radicals, leading to higher melanoma risk (76).

## 6 Therapeutic targeting HERV

Given the critical role of HERVs in cancer pathogenesis, emerging research efforts have been initiated to explore HERV-targeted antitumor therapies, current therapeutic strategies mainly involve direct HERV-targeting approaches and indirect HERV-targeting through epigenetic reprogramming.

Direct HERV-targeting approaches, aimed at suppressing HERV activation or expression, have shown promising therapeutic potential. Blocking the expression of HERVs can also exert anti-tumor effects. In TVM-A12 melanoma cells, non−nucleoside reverse transcriptase inhibitors (NNRTIs), such as nevirapine and efavirenz, effectively inhibit endogenous reverse transcriptase activity. This inhibition blocks the activation of HERV−K, thereby suppressing the expansion of the CD133+ stem−like subpopulation and inducing apoptosis in these cells (84). In prostate cancer, the application of HERVs reverse transcriptase inhibitor Abacavir reduces the growth and invasiveness of tumor through postponing cell cycle and inducing cellular senescence (85). Clinically registered cancer trials directly targeting HERV-derived antigens remain limited. One study has provided preclinical evidence supporting the targeting of the HERV-derived antigen HERV-E in clear cell renal cell carcinoma. The authors showed that HERV-E TCR-transduced T cells specifically recognized and killed HLA-A11^+^/HERV-E^+^ clear cell renal cell carcinoma (ccRCC) cells *in vitro* and induced tumor regression *in vivo*, thereby supporting clinical evaluation in metastatic ccRCC (86).

HERVs can also be targeted indirectly through epigenetic reprogramming using DNA methyltransferase inhibitors (DNMTis), such as 5-aza-CdR, which has been approved for the treatment of AML. The application of DNMTis in some cancers (e.g., colorectal cancer, ovarian cancer), can induce the activation of HERVs and stimulate the production of HERV dsRNAs, and then trigger an immune response, thereby exerting anti-tumor effects (87, 88). Low-dose 5-aza-CdR relieves epigenetic repression and promotes the accumulation of dsRNAs derived from HERVs, thereby activating MDA5–MAVS–IRF7 signaling and inducing an antiviral response (87). In some ovarian cancer cell lines, HERV silencing is controlled by DNA methylation and H3K9 methylation, serving a therapeutic target for DNMTi 5-aza-CdR and G9A, a histone methyltransferase that catalyzes mono- and di-methylation of histone H3 lysine 9, respectively. Combined 5-aza-CdR and G9A inhibition synergistically enhances HERV reactivation, increases dsRNAs, and strengthens the viral mimicry response. Importantly, the study also shows that these effects are context-dependent and that not all HERVs are equally capable of activating the viral defense pathway (88). Similarly, therapeutic targeting histone demethylase LSD1 increases HERV expression, induces dsRNAs stress, activates type I interferon (IFN-I) and stimulates the anti-tumor immunity, thereby inhibiting tumor progression (89). In ErbB2+ breast cancer, polycombcomplex 2 (PRC2)-mediated H3K27 trimethylation silences the expression of HERVs and inhibits the release of IFN-I, which may associate with the resistance to treatment of Trastuzumab monoclonal antibodies targeting ErbB2. Therapeutic targeting EZH2, which is the core enzyme of PRC2, could reverse the expression of HERVs, thereby restoring IFN signaling and significantly enhancing the efficacy of anti-ErbB2 monoclonal antibodies (90) It’s worth noting that HERVs function in a context-dependent manner, where PRC2-induced aberrant expression of HERVs is specific to tumor cells rather than healthy tissues, ensuring the therapeutic specificity of EZH2 inhibitors (90). Besides, the inherent genomic instability of tumor cells further confers their increased sensitivity, comparing to that of healthy tissues (91). In DLBCL patients with *TP53* mutation, the application of decitabine can downregulate the expression of methyltransferase SUV39H1, inhibit H3K9 trimethylation and induce HERV expression, contribute to release of interferons and activation of anti-tumor immunity. The combination therapy of decitabine and doxorubicin serves a novel therapeutic strategy for *TP53* mutation-driven drug-resistant lymphoma (92).

Epigenetic agents such as DNMTis exert broad pleiotropic effects, making it difficult to attribute their antitumor activity entirely to HERV reactivation. For example, agents such as DNMTis and LSD1 inhibitors could activate HERVs and simultaneously altering host gene expression and multiple cellular signaling pathways (93). Besides, their ability to induce HERV expression, trigger viral mimicry, and enhance downstream immune activation is context dependent, where distinct tumor types, HERV loci, and HERV families may elicit either immune-stimulatory or oncogenic effects. Future studies investigating the underlying mechanisms of HERV reactivation, as well as locus- and context-specific strategies are anticipated.

Given that HERV-modulating therapies can exert immunomodulatory effects, several studies have investigated their combination with immune checkpoint inhibitors, with encouraging results. DNMTis treatment sensitizes to anti-CTLA-4 antibodies in a preclinical model of melanomas (27). LSD1 depletion also increases the efficacy of anti-PD-1 therapy in the mouse B16 melanoma model (89). According to a small retrospective cohort (n=24) of metastatic ccRCC patients, increased intratumoral ERV3–2 expression was associated with significantly improved objective response rate and prolonged progression-free survival upon treatment with anti-PD-1/PD-L1 monotherapy (94).

## 7 Conclusion and perspective

HERVs regulate cancer development and progression through multiple oncogenic pathways, promoting cancer deterioration and metastasis. By analyzing and summarizing key genes and regulatory pathways, we can gain a comprehensive view into the pathogenic mechanisms of HERVs, thereby exploring novel diagnostic and therapeutic strategies for cancer from the perspective of HERVs.

Although it is clear that HERVs are closely associated with cancer, they cannot be uniformly defined as oncogenic factors. On the one hand, HERVs can contribute to the progression of various cancers by regulating host genes, signaling pathways, and other cellular processes. On the other hand, in certain cancer contexts, HERVs may also exert tumor-suppressive effects. Besides, the expression of most HERVs is under epigenetic regulation. Whether HERVs function as a causative driver or passenger events remains to be further investigated in the context of diseases, which may in turn facilitate the development of novel diagnostic and therapeutic strategies from the perspective of HERVs.
